# Co_3_O_4_@NiMoO_4_ composite electrode materials for flexible hybrid capacitors

**DOI:** 10.1007/s12200-022-00029-0

**Published:** 2022-05-26

**Authors:** Yongli Tong, Tengxi Zhang, Yuchen Sun, Xiaowei Wang, Xiang Wu

**Affiliations:** 1 School of Materials Science and Engineering, Shenyang University of Technology, Shenyang, 110870 China; 2 Wuhan National Laboratory for Optoelectronics, Huazhong University of Science and Technology, Wuhan, 430074 China; 3 School of Science, Shenyang Ligong University, Shenyang, 110159 China

**Keywords:** Supercapacitor, Co_3_O_4_@NiMoO_4_ nanowires, Specific capacitance, Energy density

## Abstract

Co_3_O_4_ nanomaterials as electrodes have been studied widely in the past decade due to their unique structural characteristics. However, their performance does not yet reach the level required for practical applications. It is, nevertheless, an effective strategy to synthesize hybrid electrode materials with high energy density. Herein we prepare Co_3_O_4_@NiMoO_4_ nanowires by a two-step hydrothermal method. The as-obtained sample can be directly used as cathode material of supercapacitors; with specific capacitance of 600 C/g at 1 A/g. An assembled capacitor delivers an energy density of 36.1 Wh/kg at 2700 W/kg, and retains 98.2% of the initial capacity after 8000 cycles.

**Graphical Abstract:**

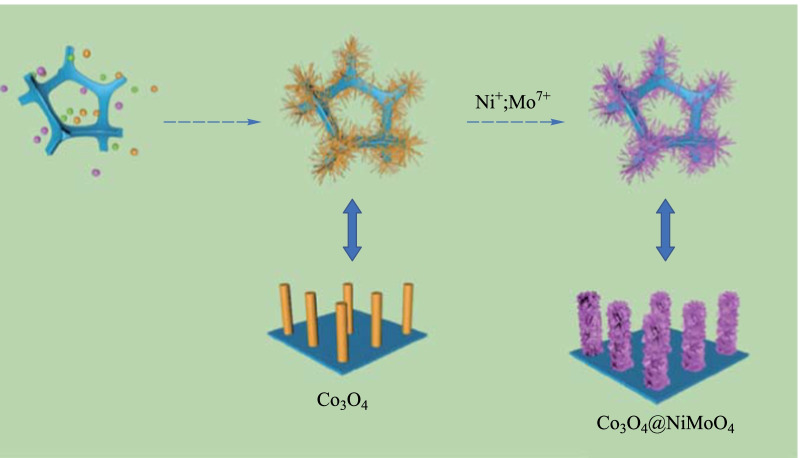

## Introduction

The shortage of fossil energy resources leads to urgent requirement for the exploration of sustainable energy conversion and storage equipment [1–3]. Among them, the supercapacitor (SC) is an excellent energy storage device due to high power density and long cycle life [4, 5]. According to energy storage mechanism, SCs can be classified into electrical double-layer capacitors and pseudo-capacitors. The latter type possesses a greater potential than the former in terms of specific capacitance, due to highly reversible redox reactions of electrode materials [6–8]. However, low energy density restricts their practical application. Therefore, it is extremely important to develop high-performance electrode materials for this type of SCs.

At present, transition metal oxides are considered to be promising candidates for SC electrode materials [9–13]. However, these traditional cathode materials still show relatively poor conductivity and low specific capacitance. The ternary transition metal oxides show better conductivity than some binary counterparts due to the multiple oxidation valence states [14–16]. Co-based materials have been used as cathodes for SCs [17–20]. It is still crucial to tailor their shapes and structures to improve the electrochemical performance by constructing a Co_3_O_4_-based hybrid structure [21–23].

Herein, we report Co_3_O_4_@NiMoO_4_ nanowire structures grown on porous nickel (Ni) foam via a two-step hydrothermal method. With conductive Ni foam as the skeleton, electrode materials with high capacitance can be obtained. The as-obtained material delivers a capacity of 600 C/g at 1 A/g. Asymmetric SCs are assembled, with Co_3_O_4_@NiMoO_4_ as cathode and activated carbon as anode (Co_3_O_4_@NiMoO_4_//AC). The device shows an energy density of 36.1 Wh/kg and long cycle stability.

## Methods

### Synthesis of Co_3_O_4_ nanowires

First, the Ni foam (2 cm × 1 cm, as substrate) was washed three times with absolute ethanol and deionized (DI) water- by ultrasonic cleaner (SK7200H, Shanghai Kedao). Then, 5 mmol CoCl_2_·6H_2_O, 10 mmol NH_4_F and 3 mmol urea were added into 60 mL DI water. The solution and one piece of cleaned Ni foam were transferred into a 100 mL autoclave and kept for 8 h at 120 °C. The as-obtained Co_3_O_4_ nanowire sample was washed with DI water and absolute ethanol, and dried for 8 h at 60 °C. Finally, this sample was calcined in a muffle furnace (KSL-1100X, Hefei Kejing) at 400 °C for 2 h at a heating rate of 2 °C/min.

### Synthesis of Co_3_O_4_@NiMoO_4_ composite

Briefly, as-prepared Co_3_O_4_ nanowires were utilized as the core structure for the growth of Co_3_O_4_@NiMoO_4_ composite. 3 mmol Ni(NO_3_)_2_·6H_2_O and 2 mmol NaMoO_4_·2H_2_O were dissolved in 60 mL deionized water. The solution and the previously obtained Co_3_O_4_ nanowires sample was kept in a reactor for 2 h at 160 °C. The final Co_3_O_4_@NiMoO_4_ composite sample was calcined in air at 400 °C for 2 h. Single NiMoO_4_ samples were obtained according to the above route without the addition of Co_3_O_4_ precursors.

### Structure characterization

X-ray diffraction (XRD) patterns of the samples were measured on a X-ray diffractometer (BRUKER D8) using Cu Kα radiation (*λ* = 1.5406 Å). Morphological and structural features were characterized through scanning electron microscope (SEM, Sigma500 field emission) and X-ray photoelectron Spectroscope (XPS, ESCALAB250).

### Fabrication of the asymmetric supercapacitor (ASC) device

Activated carbon, acetylene black and polytetrafluoroethylene (PTFE) were mixed in a mass ratio of 7:2:1. The mixture was coated on a cleaned Ni foam (2 cm × 1 cm) to be used as anode of the ASC. The synthesized Co_3_O_4_@NiMoO_4_ composite sample was used as cathode. The electrolyte was prepared as follows: 30 mL of water was poured into a beaker and heated to 95 °C with a magnetic stirrer. Then adding 3 mg PVA and 3 g KOH and stirring it until a clear solution was obtained. A separator was used to isolate the anode from the cathode. The ASC device was sealed with an aluminum-plastic film.

### Electrochemical measurements

In a three-electrode system, the electrochemical performance of the electrodes was measured in 3 mol KOH electrolyte, including cyclic voltammetry (CV), galvanostatic charge/discharge (GCD) and electrochemical impedance spectroscopy (EIS) curves. Three samples (Co_3_O_4_, NiMoO_4_, and Co_3_O_4_@NiMoO_4_) were employed as the working electrode respectively, Hg/HgO as a reference electrode, and Pt plate as a counter one. The specific capacitance (*C*_s_) of the samples can be obtained by applying discharge time (Δ*t*):

1 $$C_{{\text{s}}} = I\Delta t/m,$$

where *I* stands for current density, *m* represents the mass of the electrode.

An ASC was assembled by using the Co_3_O_4_@NiMoO_4_ electrode as the cathode and AC electrode as the anode. Energy density (*E*) and power density (*P*) can be obtained through the equations as follows:

2 $$E = {1}/{2}C_{{\text{s}}} \left( {\Delta V} \right)^{{2}} ,$$

3 $$P = {36}00E/t.$$

## Results and discussion

**Fig. 1:**
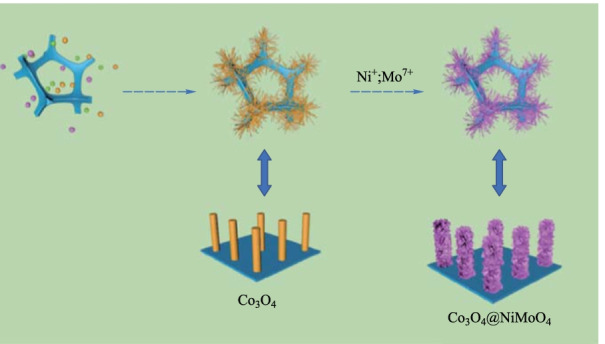
Schematic of the structure of Co_3_O_4_@NiMoO_4_ composite samples

**Fig. 2:**
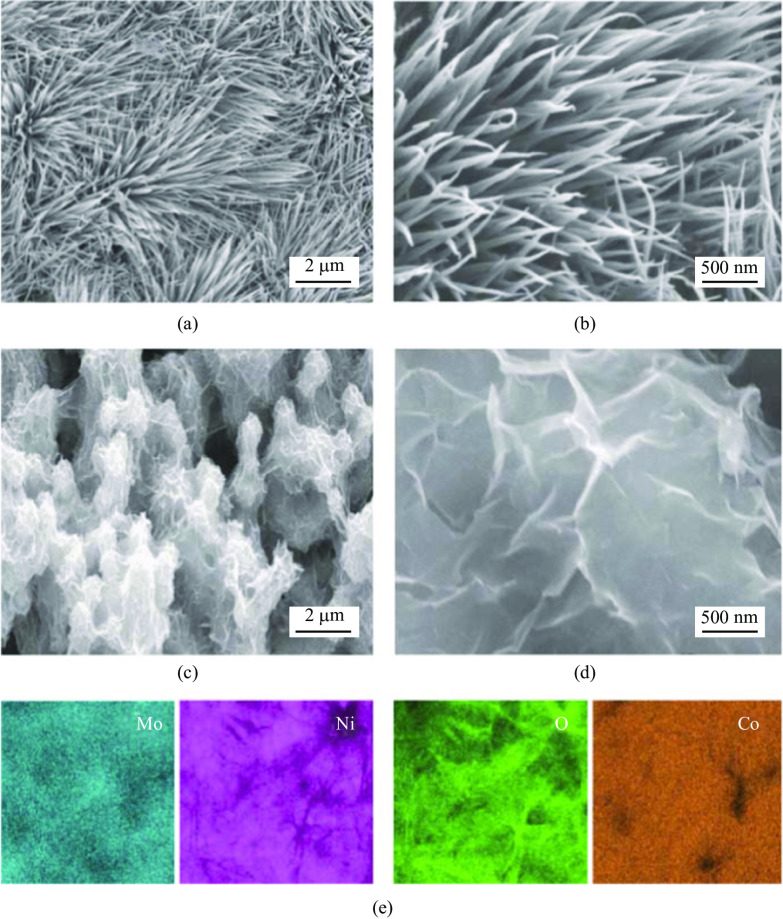
Morphology characterization. **a** and **b** Co_3_O_4_ samples. **c** and **d** Co_3_O_4_@NiMoO_4_ samples. **e** Elemental mappings

Figure 1 shows the schematic of the structure of productsCo_3_O_4_@NiMoO_4_ composite samples. Ni foam is directly used to grow Co_3_O_4_ nanowires due to its 3D conductive porous characteristics. Co_3_O_4_ samples and Co_3_O_4_@NiMoO_4_ composite samples are prepared as described in Sects. 2.1 and 2.2. Then we observed the morphologies of the samples with different magnifications using SEM. As shown in Fig. 2a, the Co_3_O_4_ sample presented a wire-like shape and was evenly covered on the Ni foam. From Fig. 2b, it can be seen that the average diameter of nanowires was 20 nm. Figure 2c and d present the morphologies of Co_3_O_4_@NiMoO_4_ composite samples, which show the NiMoO_4_ nanosheets were coated on the surface of Co_3_O_4_ nanowires, forming a core–shell structure as illustrated in Fig. 1. The surface of nanosheet becomes obviously rough. Figure 2e shows that the four elements of Co, Ni, Mo and O were evenly distributed on the surface of the sample.

**Fig. 3:**
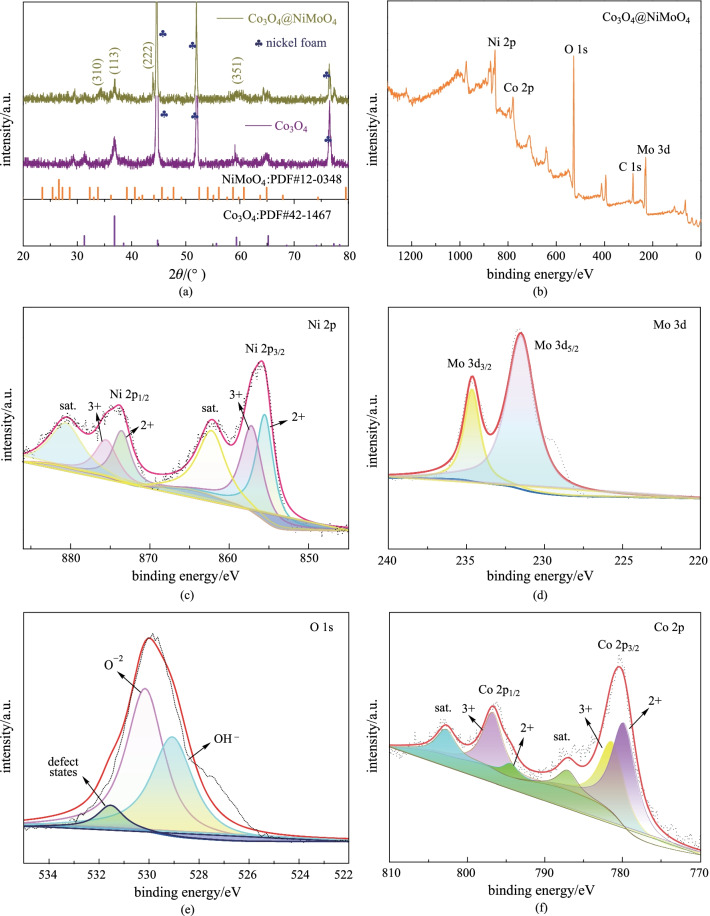
**a** XRD patterns of the samples. **b** Full spectra of XPS spectra of Co_3_O_4_@NiMoO_4_. Samples **c** Ni 2p, **d** Mo 3d, **e** O1s, and **f** Co 2p

The XRD patterns of Co_3_O_4_@NiMoO_4_ samples are shown in Fig. 3a. The peaks at 18.95°, 55.27° and 59.28° correspond to (111), (422) and (511) of Co_3_O_4_ phase (JCPS: 42-1467). The peaks at 31.14°, 36.69° and 64.98° can be assigned to (220), (311) and (440) of NiMoO_4_ (JCPS: 12-0348). It is demonstrated that NiMoO_4_ nanosheets were successfully grown on the surface of the Co_3_O_4_ nanowires. XPS was used to further study the surface chemical states of Co_3_O_4_@NiMoO_4_ composite sample. The survey spectra (Fig. 3b) indicated that the Co_3_O_4_@NiMoO_4_ sample contained Co, Ni, Mo and O elements. Ni 2p spectra (Fig. 3c) shows four peaks at 855.4, 873.4 and 857.1, 875.6 eV, which are attributed to Ni^2+^ and Ni^3+^. In addition, two satellite peaks at 862.1 and 880.4 eV can be assigned to the high oxidation state [24]. Mo 3d peaks can be split into two peaks of Mo 3d_5/2_ and Mo 3d_3/2_, as shown in Fig. 3d. The peak binding energy at 231.6 eV belongs to Mo 3d_5/2_. However, the peak at 234.7 eV is from Mo 3d_3/2_, which further confirm the existence of Mo^6+^ oxidation state [25]. In Fig. 3e, O 1 s peaks at 531.8, 530.6 and 529.4 eV correspond to defect oxygen, O^2−^ and OH^−^ , respectively [26]. In Fig. 3f, two spin-orbital doublet peaks are well fitted to Co 2p_1/2_ and Co 2p_3/2_, revealing that Co^2+^ and Co^3+^ co-exist in the as-prepared composite material. Moreover, the peaks at 786.1 and 804.5 eV present the shakeup satellites [19].

**Fig. 4:**
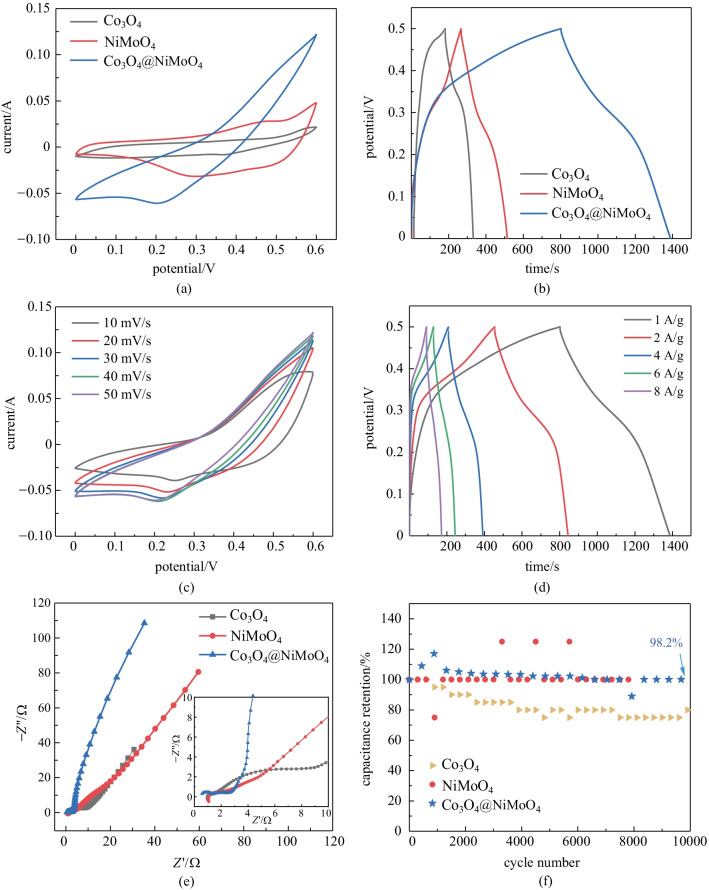
**a** CV curves at 50 mV/s. **b** GCD curves at 1 A/g. **c** CV curves. **d** GCD curves. **e** Nyquist plots. **f** Cycle performance

Figure 4a shows CV curves of the Co_3_O_4,_ NiMoO_4_, Co_3_O_4_@NiMoO_4_ samples at scan rate of 50 mV/s. The obvious redox peaks suggest that the samples possess pseudo-capacitive characteristics. The Co_3_O_4_@NiMoO_4_ samples show the largest integral area, which means that hybrid samples present excellent electrochemical performance. Co_3_O_4_@NiMoO_4_ samples present the longest discharge time (Fig. 4b), revealing their maximal specific capacitance. It can be calculated by Eq. (1) that the Co_3_O_4_@NiMoO_4_ sample possesses the specific capacitance of 600 C/g, which is higher than those of Co_3_O_4_ (177.9 C/g) and NiMoO_4_ (315 C/g). The enhanced performance can be attributed to the synergistic effect between two individual materials. On one hand, the electrical conductivity can be improved and the transmission of electrons and ions can be facilitated. On the other hand, Co_3_O_4_ is a p-type semiconductor. As the core material, it undergoes inter-band transition to form electron–hole pairs, which result in strong redox ability. A weak electric field is formed between the two composite materials, which can prevent the recombination of electrons and holes, thus greatly improving the electrochemical performance.

**Fig. 5:**
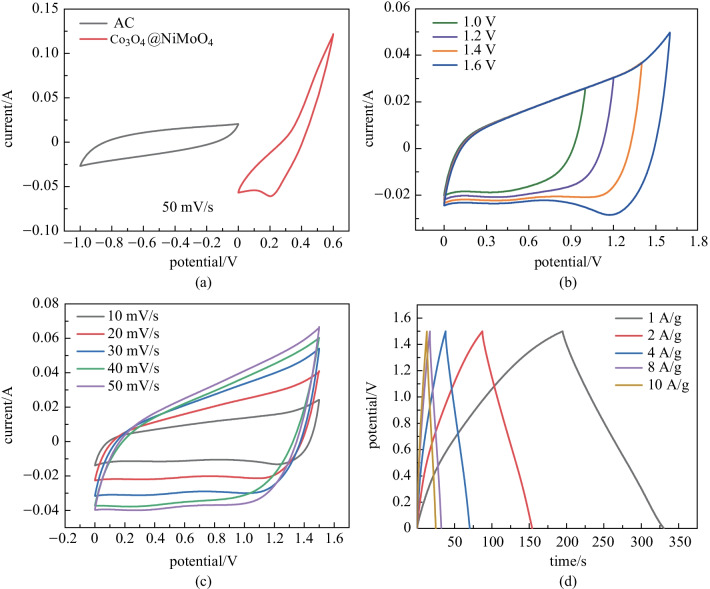
**a** CV curves of Co_3_O_4_@NiMoO_4_//AC ASC device at 50 mV/s. **b** CV curves at different potential windows. **c** CV curves at different scan rates. **d** Charge–discharge curves at different current densities

**Fig. 6:**
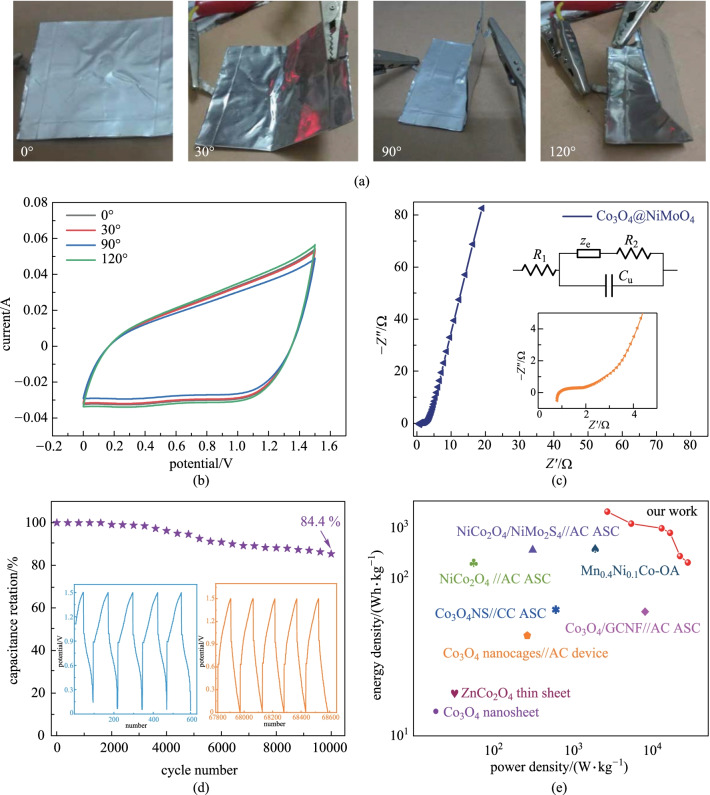
**a** Digital photos of the flexible device. **b** CV curves at various bending angles at the same scan rate. **c** Nyquist plots. **d** Cycling stability. **e** Ragone plots

Figure 4c shows the CV curves of the prepared Co_3_O_4_@NiMoO_4_ sample at different scan rates. The curve shapes further reveal a pseudo-capacitance behavior. Even at scan rates of 50 mV/s, the initial shape of CV curves is still unchanged, which confirms that the composite samples possess an excellent electrical conductivity and high-rate performance. The corresponding GCD curves are shown in Fig. 4d. Even at the current density of 8 A/g, the specific capacitance can reach 97% of initial value.

Figure 4e shows typical Nyquist plots of the three samples. The Co_3_O_4_@NiMoO_4_ electrode possesses the lowest resistance of about 0.5 Ω in the three samples. To evaluate the cycle stability of the samples, the long cycle measurements were conducted at current density of 1 A/g. The results are shown in Fig. 4f. The Co_3_O_4_@NiMoO_4_ composite sample shows the 98.2% capacitance retention after 10000 cycles.

The performances of the fabricated ASC device were also measured. The CV curves of the device are shown in Fig. 5a. Co_3_O_4_@NiMoO_4_ and activated carbon electrodes possess the potential window from 0 to 0.6 V and − 1 to 0 V, respectively. From Fig. 5b, it can be found that the stable voltage window of ASC is 0–1.6 V. The Co_3_O_4_@NiMoO_4_//AC device (Fig. 5c) shows the voltage windows from 0 to 1.6 V at scan rates from 10 to 50 mV/s. GCD measurement (Fig. 5d) is conducted at different current densities with a voltage window of 1.5 V. It clearly shows that the device has a long discharge time.

The mechanical stability of energy storage devices is important for flexible electronic products. The mechanical stability of the fabricated ASC device was further investigated, as shown in Fig. 6a. When the device was folded at 30°, 90° and 120°, the shapes of the CV curves remains unchanged (Fig. 6b), revealing its outstanding stability and flexibility. It could be ascribed to the flexibility of the Ni foam and the tight contact between the electrode material and the Ni substrate. The EIS curves of the device are presented in Fig. 6c, revealing a low equivalent resistance and fast electron transfer rate. The lower inset is local EIS curve and the upper inset shows the corresponding equivalent circuit. Cycle performance (Fig. 6d) is a key performance for the application of SCs. The result demonstrates that 84.4% of the initial specific capacitance can be retained after 10000 cycles, indicating that the Co_3_O_4_@NiMoO_4_//AC device possesses an excellent electrochemical stability. From the CV curves of the first five cycles and the last five cycles, it can be found that the charging and the discharging time are always symmetric, indicating that the device presents very good reversibility and high-rate performance. Moreover, the discharge time of the last five cycles does not reduce compared with that of the first five cycles. which further confirms that there is little drop of the capacitance over 10000 cycles. Figure 6e is a Ragone plot of several devices. Table 1 shows the electrochemical performance of the devices based on different electrode materials. It was found that the Co_3_O_4_@NiMoO_4_//AC device can deliver an energy density of 36.1 Wh/kg at 2700 W/kg, which is higher than those reported in previous literatures [27–34].

**Table 1:** Electrochemical performance comparison of several SC devices based on transition metal oxides

Material	Energy density/(Wh⋅kg^−1^)	Power density/(W·kg^−1^)	Reference
NiCo_2_O_4_@NiMo_2_S_4_	30.7	374.9	[27]
Co_3_O_4_	26.6	189.5	[28]
Co_3_O_4_ nanohorn	31.7	16.7	[29]
NiCo_2_O_4_	27.4	493.2	[30]
Co_3_O_4_ nanocages	19.8	90.2	[31]
Mn_0.4_Ni_0.1_Co-OA	32.2	770.2	[32]
Co_3_O_4_ thin sheets	8.0	0.8	[33]
ZnCo_2_O_4_ sheets	9.78	33.98	[34]
Co_3_O_4_@NiMoO_4_	36.1	2700	This work

## Conclusion

In summary, the core–shell Co_3_O_4_@NiMoO_4_ samples were successfully grown on Ni foam by a simple hydrothermal route. The synthesized samples presented an excellent specific capacitance (600 C/g at 1 A/g) and cycle stability. After 10000 charge–discharge cycle tests, the capacitance retention of the as-prepared composite still reached 98.2%, which shows long-term charging and discharging behavior. The as-assembled ASC delivered superior electrochemical performance with an energy density of 36.1 Wh/kg at 2700 W/kg and 84.4% initial capacity retention after 10000 cycles.

## References

[1] Liu Y, Wu X (2021). Hydrogen and sodium ions co-intercalated vanadium dioxide electrode materials with enhanced zinc ion storage capacity. Nano Energy.

[2] Shi W, Lv X, Shen Y (2018). BiOI/WO_3_ photoanode with enhanced photoelectrochemical water splitting activity. Front. Optoelectron..

[3] Liu Y, Hu P, Liu H, Wu X, Zhi C (2020). Tetragonal VO_2_ hollow nanospheres as robust cathode materials for aqueous zinc ion batteries. Mater. Today Energy.

[4] Zhao Y, He J, Dai M, Zhao D, Wu X, Liu B (2020). Emerging CoMn-LDH@MnO_2_ electrode materials assembled using nanosheets for flexible and foldable energy storage devices. J. Energy Chem..

[5] Dai M, Liu H, Zhao D, Zhu X, Umar A, Algarni H, Wu X (2021). Ni foam substrates modified with a ZnCo_2_O_4_ nanowire coated with Ni(OH)_2_ nanosheet electrode for hybrid capacitors and electrocatalysts. ACS Appl. Nano Mater..

[6] Liu H, Zhao D, Liu Y, Tong Y, Wu X, Shen G (2021). NiMoCo layered double hydroxides for electrocatalyst and supercapacitor electrode. Sci. China Mater..

[7] Silvia PJ, Eddington KM, Harper KL, Burgin CJ, Kwapil TR (2021). Reward-seeking deficits in major depression: unpacking appetitive task performance with ex-Gaussian response time variability analysis. Motivation Sci..

[8] Zhao D, Dai M, Liu H, Zhu X, Wu X (2021). PPy film anchored on ZnCo_2_O_4_ nanowires facilitating efficient bifunctional electrocatalysis. Materials Today Energy.

[9] Tong Y, Cheng X, Liu X, Qi D, Chi B, Wang Y (2020). Hybrid Co_3_O_4_/Co_9_S_8_ nanowires for high-performance asymmetric supercapacitors. J. Nanoelectron. Optoelectron..

[10] Liu C, Wu X, Wang B (2020). Performance modulation of energy storage devices: a case of Ni-Co-S electrode materials. Chem. Eng. J..

[11] Liu H, Zhao D, Liu Y, Hu P, Wu X, Xia H (2019). Boosting energy storage and electrocatalytic performances by synergizing CoMoO_4_@MoZn_22_ core-shell structures. Chem. Eng. J..

[12] Liu H, Zhao D, Hu P, Chen K, Wu X, Xue D (2020). Design strategies toward achieving high-performance CoMoO_4_@Co_1.62_Mo_6_S_8_ electrode materials. Mater. Today Phys..

[13] Peng S, Li L, Wu H, Madhavi S, Lou X (2015). Controlled growth of NiMoO_4_ nanosheet and nanorod arrays on various conductive substrates as advanced electrodes for asymmetric supercapacitors. Adv. Energy Mater..

[14] Qiu K, Lu Y, Zhang D, Cheng J, Yan H, Xu J, Liu X, Kim J, Luo Y (2015). Mesoporous hierarchical core/shell structured ZnCo_2_O_4_/MnO_2_ nanocone forests for high-performance supercapacitors. Nano Energy.

[15] Zhao D, Liu H, Wu X (2019). Bi-interface induced multi-active MCo_2_O_4_@MCo_2_S_4_@PPy (M=Ni, Zn) sandwich structure for energy storage and electrocatalysis. Nano Energy.

[16] Gao X, Zhang Y, Huang M, Li F, Hua C, Yu L, Zheng H (2014). Facile synthesis of Co_3_O_4_@NiCo_2_O_4_ core–shell arrays on Ni foam for advanced binder-free supercapacitor electrodes. Ceram. Int..

[17] Gu Z, Guo J, Zhao X, Wang X, Xie D, Sun Z, Zhao C, Liang H, Li W, Wu X (2021). High-ionicity fluorophosphate lattice via aliovalent substitution as advanced cathode materials in sodium-ion batteries. InfoMat.

[18] Dai M, Zhao D, Wu X (2020). Research progress on transition metal oxide based electrode materials for asymmetric hybrid capacitors. Chin. Chem. Lett..

[19] Liu H, Dai M, Zhao D, Wu X, Wang B (2020). Realizing superior electrochemical performance for asymmetric capacitors through tailoring electrode architectures. ACS Appl. Energy Mater..

[20] Dai M, Zhao D, Liu H, Tong Y, Hu P, Wu X (2020). Nanostructure and doping engineering of ZnCoP for high performance electrolysis of water. Mater. Today Energy.

[21] Zhu X, Meng F, Zhang Q, Xue L, Zhu H, Lan S, Liu Q, Zhao J, Zhuang Y, Guo Q, Liu B, Gu L, Lu X, Ren Y, Xia H (2021). LiMnO_2_ cathode stabilized by interfacial orbital ordering for sustainable lithium-ion batteries. Nat. Sustain..

[22] Xing L, Dong Y, Hu F, Wu X, Umar A (2018). Co_3_O_4_ nanowire@NiO nanosheet arrays for high performance asymmetric supercapacitors. Dalton Trans. (Cambridge, England).

[23] Tong Y, Liu H, Dai M, Xiao L, Wu X (2020). Metal-organic framework-derived Co_3_O_4_/PPy bifunctional electrocatalysts for efficient overall water splitting. Chin. Chem. Lett..

[24] Xing L, Dong Y, Wu X (2018). Hierarchical Co_3_O_4_@Co_9_S_8_ nanowall structures assembled by many nanosheets for high performance asymmetric supercapacitors. RSC Adv..

[25] Hu P, Liu Y, Liu H, Xiang W, Liu B (2021). MnCo_2_O_4_ nanosheet/NiCo_2_S_4_ nanowire heterostructures as cathode materials for capacitors. ACS Appl. Nano Mater..

[26] Tong Y, Dai M, Xing L, Liu H, Sun W, Wu X (2020). Asymmetric hybrid capacitor based on NiCo_2_O_4_ nanosheets electrode. Wuli Huaxue Xuebao.

[27] Zhao D, Dai M, Liu H, Chen K, Zhu X, Xue D, Wu X, Liu J (2019). Sulfur induced interface engineering of hybrid NiCo_2_O_4_@NiMo_2_S_4_ structure for overall water splitting and flexible hybrid energy storage. Adv. Mater. Interfaces.

[28] Lu Y, Deng B, Liu Y, Wang J, Tu Z, Lu J, Xiao X, Xu G (2021). Nanostructured Co_3_O_4_ for achieving high-performance supercapacitor. Mater. Lett..

[29] Sivakumar P, Jana M, Kota M, Jung MG, Gedanken A, Park HS (2018). Controllable synthesis of nanohorn-like architectured cobalt oxide for hybrid supercapacitor application. J. Power Sources.

[30] Zhao D, Hu F, Umar A, Wu X (2018). NiCo_2_O_4_ nanowires based flexible electrode materials for asymmetric supercapacitors. N. J. Chem..

[31] Zhang H, Yan B, Zhou C, Wang J, Duan H, Zhang D, Zhao H (2021). MOF-derived hollow and porous Co_3_O_4_ nanocages for superior hybrid supercapacitor electrodes. Energy Fuels.

[32] Liang S, Wang H, Li Y, Qin H, Luo Z, Chen L (2021). Ternary synergistic transition metal oxalate 2D porous thin sheets assembled by 3D nanoflake array with high performance for supercapattery. Appl. Surf. Sci..

[33] Jiang Y, Chen L, Zhang H, Zhang Q, Chen W, Zhu J, Song D (2016). Two-dimensional Co_3_O_4_ thin sheets assembled by 3D interconnected nanoflake array framework structures with enhanced supercapacitor performance derived from coordination complexes. Chem. Eng. J..

[34] Song D, Zhu J, Li J, Pu T, Huang B, Zhao C, Xie L, Chen L (2017). Free-standing two-dimensional mesoporous ZnCo_2_O_4_ thin sheets consisting of 3D ultrathin nanoflake array frameworks for high performance asymmetric supercapacitor. Electrochim. Acta.

